# Health Impacts from Corn Production Pre-and Post-NAFTA Trade Agreement (1986–2013)

**DOI:** 10.3390/ijerph13070709

**Published:** 2016-07-13

**Authors:** Oliver Mendoza-Cano, Ramón Alberto Sánchez-Piña, Álvaro Jesús González-Ibarra, Efrén Murillo-Zamora, Cynthia Monique Nava-Garibaldi

**Affiliations:** 1 Center for Health and the Global Environment, Department of Environmental Health, Harvard TH Chan School of Public Health, Boston, MA 02215, USA; rsanchez@hsph.harvard.edu (R.A.S.-P.); alvaro.goniba@gmail.com (A.J.G.-I.); 2 Facultad de Ingeniería Civil, Universidad de Colima, Coquimatlán 28400, Mexico; cynthia.nava20@gmail.com; 3 Instituto Mexicano del Seguro Social, Unidad de Medicina Familiar 19. Colima, Colima 28000, Mexico; efren.murilloza@imss.gob.mx

**Keywords:** life cycle assessment, corn, DALYs

## Abstract

Life cycle assessment (LCA) is a powerful methodology for the study of health impacts and public policies. We performed this study to quantitatively explain the potential health impacts on disability-adjusted life years (DALYs) of corn produced in Mexico and imported from the United States of America (U.S.) from 1984 until 2014. The processes are hybrid and organic corn production. The functional unit was defined as 1 ton of corn production. Results indicate a total value of 178,431, 244,175, and 283,426 DALYs of three decades: 1984–1993, 1994–2003, and 2004–2013, of Mexican production; the U.S. production and transport were also calculated, showing values of 29,815, 65,837, and 107,729 for the same three decades. Additionally, DALYs were obtained for the category of human health and climate change by functional unit: 802.31 (1984–1993), 802.67 (1994–2003), and 803.92 (2004–2013), and for imported corn transported to Mexico from the U.S., 859.12 (1984–2013). DALYs on human toxicity were obtained: 99.05 (1984–1993), 99.05 (1994–2003), and 99.04 (2004–2013), and for the corn imported and transported to Mexico from the U.S., 116.25 (1984–2013). Conclusions: Environmental and health impacts in terms of DALYs are higher when corn is imported versus the corn produced in Mexico. Environmental health and nominal corn cultivation and transport impacts have increased as a result of the North American Free Trade Agreement (NAFTA). Mexico needs to redefine its public policies to suffer less of an environmental burden from corn to ensure global environmental health and food security.

## 1. Introduction

Corn is the second most cultivated crop with the highest production worldwide with 615,533,645 Mton (millions of tons) [1]. The U.S. is the largest producer with 273,820,066 Mton, while Mexico produces 22,069,254 Mton. Corn is also the main crop and food base of Mexico, with 27% of agricultural land, comprised of 2.8 million Hectares of corn farmlands [2]. During the period of 1996–2006, the cultivation of corn occupied 51% of all cultivated and harvested lands, generating 7.4% of the total agricultural production, and representing 30% of the total value of production [3].

In recent years, there has been a growing concern about the sustainability of agricultural and food systems and the unforeseen effects on environment and human health [4]. Little has been done about the subject of NAFTA in relationship with the health and environmental impacts between both countries. For example, studies show that, for the U.S., the increase of exports to Mexico due to NAFTA represents 1% of its total corn production, with its associated environmental impacts, like the use of chemicals, water contamination due to runoff, unsustainable use of water for irrigation, the expansion of genetically modified corn, soil erosion and biodiversity loss. The main environmental impact for Mexico is the threat to agro biodiversity; the low prices of imported corn is causing a decrease in native corn production, which adds to the negative environmental effects from both sides of the border. This could have deeper impacts if the loss of agro diversity in Mexico is significant [5].

Since the implementation of NAFTA, the corn imports to Mexico from the U.S. have tripled, the price of the grain has lowered almost 50%, and between 2.5 and 3 million Mexican farm workers are facing increasing economic pressure [6]. Even though there is evidence about the negative effects between both nations regarding the growing corn importation, what is unknown are the global effects of NAFTA between both countries. The U.S. is the main exporter of corn to Mexico with 99.98%, followed by small quantities from Argentina, Brazil, Chile and Guatemala [7]. The corn studies in Mexico have been focused on a small scale, especially to small farmers—“those who lose with NAFTA”—that were excluded from support for production and market. Recently, most of the attention has been directed to those on a larger scale who have become the internal and principal providers of corn [8].

There is background information about life cycle assessment (LCA) and corn production in the U.S. In those studies, the environmental performance of the grain and stubble was evaluated by the location of the corn due to crop management, soil properties, and climatic conditions [9]; however, the global environmental impacts were not quantified. Until this day, the global effects that corn production entails, before and after the NAFTA agreement, are unknown.

In this article, we calculate, nominally, the effects on health, damages to the ecosystem, and the entailed resources, with a functional unit of 1 × 10^16^ tons of corn produced in Mexico and imported from the U.S. during the last three decades (1984–2013). The LCA allows us to quantify and identify the variables that are affecting the environment. Calculating corn production by a functional unit allows us to identify the normalized environmental impacts for this crop and to have a baseline for further LCA studies and better practices.

Our results underscore the particular attributes of corn production with its environmental impact in Mexico. Analyzing several types of corn; hybrid with chemicals and organic produced in Mexico, and hybrid corn that enters Mexico from the U.S. Our expectation is that this type of knowledge will contribute not only to new research about agricultural impacts that will benefit food safety in Mexico, but also for a better policy agenda that will be able to respond to future challenges in the world.

## 2. Materials and Methods

### 2.1. Impact Evaluation

This research followed the guidelines of the life cycle evaluation developed by the International Organization for Standardization (ISO) in the series ISO 14040 to 14044 [10,11]. The environmental, health and biodiversity impacts are calculated by the ReCiPe methodology [12], Endpoint H, and the World H/H by the software SIMA PRO S version 8 (PRé Consultants bv, Amesfoort, The Netherlands). The categories considered for the impact evaluation were the general categories of human health, ecosystems, and resources. Additionally, the particular categories for functional unit analysis were also obtained, which includes climate change, human health, ozone depletion, human toxicity, photochemical oxidant formation, particulate matter formation, ionizing radiation, climate change ecosystems, terrestrial acidification, freshwater eutrophication, terrestrial ecotoxicity, freshwater ecotoxicity, marine ecotoxicity, agricultural land occupation, urban land occupation, natural land transformation, metal depletion, and fossil depletion.

### 2.2. Description of the Analyzed Processes

An environmental profile of the three decades of corn production in Mexico was calculated: 1984–1993 (pre NAFTA), 1994–2003 (post NAFTA), and 2004–2013 (current situation). The proportions of hybrid corn, cultivated with chemicals, and organic corn were estimated with values proportioned for the Commission for Environmental Cooperation [13]. Estimations were taken from the VII Mexican Agricultural Census of 1991 to propose the distribution of the use of hybrid seeds by state. This distribution per state was then multiplied by the percentage using chemical fertilizers to obtain this data. This percentage was obtained for each Mexican state from statistics of the Agro-food and Fishing Information Service (SIAP for its acronym in Spanish) of the years 2011 and 2013 in the section: “Planted surface and harvest by Mexican federal entity, according to the use of chemical fertilizers”. An estimate of organic production by each Mexican state during all of the periods of national study was calculated by obtaining the difference between the entire hybrid corn production and the one that uses chemicals in crops (Appendix A); the difference being 26.94% of the organic corn produced in Mexico. A table by functional unit was then elaborated (see Appendix B). For this, we obtained the data of corn production in Mexico from the Ministry of Agriculture, Ranching, Rural Development, Fisheries, and Food Supply of Mexico [7].

The effects of U.S. production and its transport to Mexico were also evaluated. To obtain this, three states from the U.S. were selected to estimate all corn produced and transported to Mexico: Iowa, Illinois, and Indiana, which are located in the middle of the “corn golden belt of the U.S.” (see Table 1). To estimate the quantities that are imported to Mexico, percentages were taken and calculated by quantities and locations [14], assuming the following distribution for Mexican entries: Nuevo Laredo (73%), Matamoros (7%), and Veracruz (20%). Distances were considered on Table 1 for the analysis of imported corn from the U.S. to the center of Mexico and 1133 kilometers by train for hybrid corn produced in Mexico to be processed in Mexico. For this, the mean distance between the states with higher production and industrialization were considered (Sinaloa and Nuevo Leon, respectively) to the center of the country and 100 kilometers by truck for the non-hybrid, chemical-free corn. We consider the organic corn as a self-consumption item; thus, no transportation was considered for organic corn. Table 2 shows corn data, in tons, that were considered in the evaluation.

For the water route, the kilometers by rail to the port of St. Louis, Missouri, and 2300 km by water to the port of Veracruz are considered. For the terrestrial route, the kilometers from the geographic center of each American state in a straight line to the corresponding entry point were considered: Nuevo Laredo or Matamoros, and from those points to the center of Mexico, at Mexico City. The total quantities of corn, in millions of tons, are denominated *Absolute Data,* and their impacts in health are called Total Values.

### 2.3. Analysis per Functional Unit

A product system is a set of energy connections, material, and unitary processes performing one or many defined functions [10]. For this study, the impact data from the last three decades of corn produced in Mexico and imported from the U.S., from 1984 until 2014 is grouped and normalized by functional unit (FU), which is defined as: “1 ton of corn grain produced”, and is analyzed by Endpoint (H), worldwide normalization (H/H).

### 2.4. Limitations of the System

We have excluded in the analysis, due to the lack of reliable data, the requirements of the corn production infrastructure. The normalization of the results is performed by the total absolute quantification (without normalization) of the three decades considered in this study.

## 3. Results

### 3.1. Inventory of the Life Cycle: Results from Absolute Data

**Figure 1:**
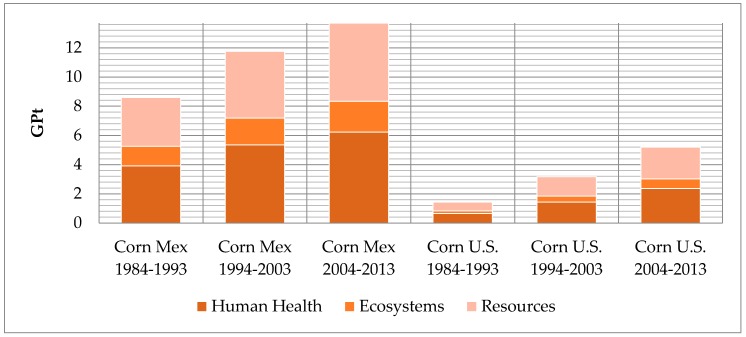
Comparison of product station by the ReCiPe Endpoint (H) V1.10/World ReCiPe H/H/single-score method.

In our analysis presented in Figure 1 (in GPt), are the three impact categories described on Table 3: Human Health, Ecosystems, and Resources. The production in Mexico and the last three decades of imports are observed and found in the same graphic.

### 3.2. Results by Functional Unit

**Figure 2:**
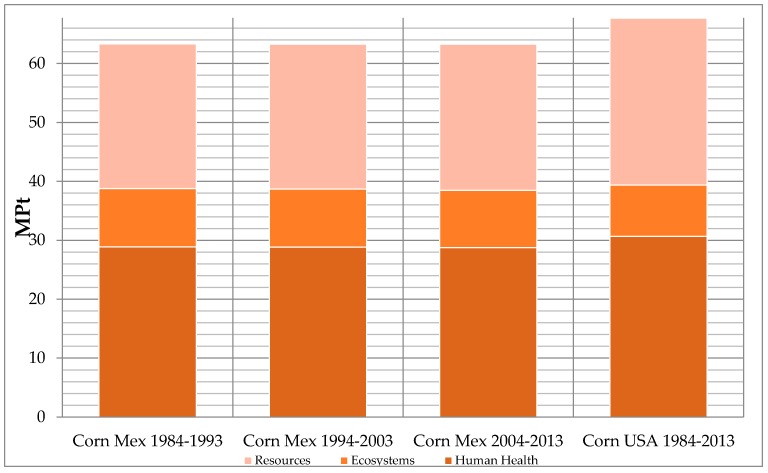
Life cycle analysis normalized by functional unit by the ReCiPe Endpoint (H) V1.10/World ReCiPe H/H/single-score method.

**Figure 3:**
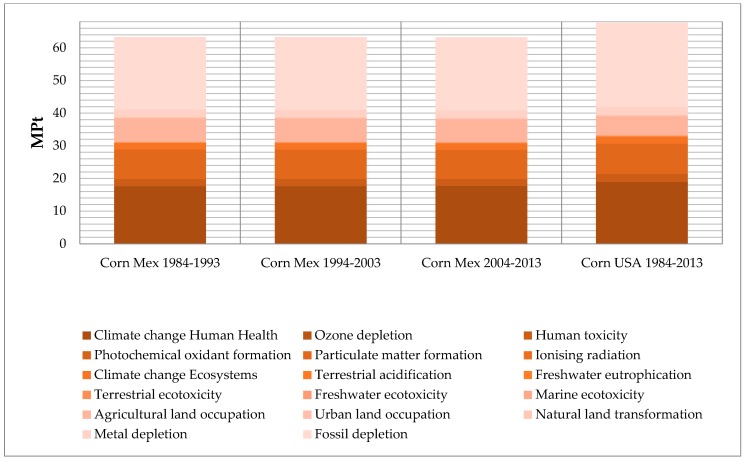
MP Graph by impact category normalized by functional unit by the ReCiPe Endpoint (H) V1.10/World ReCiPe H/H/single-score method.

The total impact values by functional unit of Mexican-produced corn, and that imported from the U.S., are presented on Table 4. The data of results by functional unit and the data-by-impact category is shown in Figure 2 and Figure 3 respectively. For the data-by-impact category there is a total value of (63.30, 63.29, and 63.26) milipoints (MP) for each of the last three decades of Mexican production, while the U.S. production and its transportation is 67.71 MP.

The impacts per functional unit of the production in Mexico remained the same for all three decades due to the assumption, according to the authors, that for 1 ton of corn produced in Mexico, the proportion of production remains the same for the following: hybrid corn, non-hybrid corn using chemicals, and organic corn. On the other hand, according to the results on hybrid corn exported from the U.S. to Mexico, the impacts remain the same for the three decades, since the corn that was imported was hybrid only (plus its transport to Mexico), and the functional unit also being 1 ton of hybrid corn.

## 4. Discussion

The results show two main findings:

1. The total values of corn production were quantified for each impact; corn is the base of Mexican food and provides food security to this country. The maximum in the last decade of corn cultivation in Mexico was found to be 13.6 GPt. This data and analysis is a baseline and reference to quantify the impacts of other established crops in Mexico. We did not find any other documented analysis in Mexico of crop life cycle to date. Sugar cane is still the highest cultivated crop worldwide and is followed closely by corn, then by other crops, such as sorghum, oranges, wheat, banana, tomato, and others. Therefore, corn studies are significant for places like Latin America where corn is the main food supply.
2. The analysis regarding functional units shows that the environmental impact representing the imports is 5 MP over the maximum values of corn that can be produced in Mexico (see Figure 2). This indicates that imported corn causes more damage to health, environment, and resources than national production and its internal transportation would entail. Most cultivated corn in Mexico is “white” and it is utilized to produce tortillas and other food products for direct human consumption. This contrasts with the “yellow” hybrid corn that is produced in the US and imported to Mexico, being the only source of imported yellow corn [15,16]. The yellow corn is commonly used as a feed grain in beef cattle diets throughout the US [17,18]. According to [19], 90 to 95 percent of cornstarch is produced using corn imported from the U.S. Tortillas are made with this cornstarch. There is no evidence of increased digestibility or nutritional value of white or yellow corn, but consumers may have resistance to accept yellow corn due to quality problems. This is also due to the knowledge that, over the years, cumulative investment in corn improvement research has been far greater for yellow corn because it is the dominant germplasm adapted to temperate environments in the developed world [20]. The ReCiPe endpoint impact through 1984–2013 in Mexico have remained on the same level because the functional unit is 1 ton of production. However, for hybrid corn exported from the U.S. to Mexico in the three decades, an increase of impacts due to transportation is observed. Transport has a very significant global impact.

One limitation of this study is the missing quantification of the yield. Regarding the strength of the findings, there is data that indicates that white corn is still dominating the production of corn grain in Mexico (national is 91% white, 8.5% yellow, and 0.5% in the other two categories) [21]. This is significant given the health implications that the importation of yellow corn had since 1984 and by the nature of imported yellow corn, which is conceived for another market not specifically for human consumption. Since NAFTA was introduced, we found that, by functional unit, the impacts are lessened by producing corn in Mexico than to produce and transport the imported corn from the U.S.; additionally, it was found that the production, by functional unit, in Mexico is more sustainable than importing the corn from the U.S. It is clear that transporting corn from large distances has a substantial impact. The key political questions are now focused on how to reduce these impacts utilizing a variety of taxes, incentives, and mechanisms for corn in Mexico. It will be necessary to make sure that new political reforms are promoted that push for healthy food production, while externalities in agriculture and food push to be lessened every day. This also implies that the impacts of different food products that are commonly used in Mexico and Latin America can be performed in future studies. Furthermore, different cultivation process/transportation methods can be proposed to improve better practices in agriculture.

There are also some social and public policy implications that should be considered due to the findings in this study. These findings are supported by the interest of the Mexican country to reduce corn importations, and to cater its own consumption with national production in the years to come. For this, it is necessary to increase corn production, giving the producers access to appropriate technologies, including better seeds [22]. There is great aptitude to cultivate corn for human consumption in Mexico. For example, 63.1% of the national surface presents some grade of suitability for corn production [23]. The majority of corn production units in Mexico are at the small and medium scale, and they operate in less than 50% of their full potential. Meanwhile, there is evidence that suggests that Mexico could increase its annual production from 23 to 33 million tons in a period of 10–15 years, which would eliminate the annual deficit of 10 million tons annually [24].

The changes in agricultural policies impact directly on dietary patterns and health-related conditions. Corn production in the U.S. is subsidized in order to produce, among others, high-fructose syrup and hydrogenated fats that are widely used in the making of processed food products [25]. A positive correlation between agricultural subsidies and the prevalence of obesity has also been observed [26]. In Latin America, changes in the production of corn have been associated negatively with cardiovascular health [27] and other diet-related chronic diseases [28].

Regarding the sustainable background of NAFTA in agriculture, we have to understand that the agricultural liberalization is strategic and important from an environmental perspective: no other sector exhibits as close a relationship with the environment as terrestrial farming. Part of the results in this paper show that categories like climate change and human health, particulate matter formation, agricultural land occupation, and fossil depletion have the largest impact by functional unit analysis. NAFTA has contributed directly to a 1–2 percent increase in annual gross emissions of carbon monoxide and sulfur dioxide due to changes in the petroleum, base metals, and transportation equipment sectors [29].

Increased corn exports from the U.S. resulted in an increase of 77,000 tons of nitrogen, phosphorus, and potassium-based loadings to U.S. waterways, with concentrated emissions in the already heavily-polluted Mississippi River Delta [30,31].

With the argument of the need for food security, NAFTA was permitted by the Mexican government. Fees were forgone and corn was imported practically without protection. Fees were not reinstated until the year 2000 [32,33]. To constrain the cost of corn, during the past two decades, the government created free trade programs and restricted imports to maintain the high domestic market price [34]. In this situation, if done rapidly, the liberalization of the agricultural trade could disrupt Mexico’s rural labor market [35,36]. If Mexico eliminates farm supports and allows import competition, Mexico’s corn sector would collapse, which will make the low-income communities and the small farmers who cultivate corn for self-consumption the most vulnerable.

## 5. Conclusions

The most probable scenario for the immediate future is that nothing will change because NAFTA is still active and it will not represent any risk for the food sovereignty of the US by exporting 1% of its production to Mexico. However, some changes could be proposed, such as those caused by an energy or petroleum crisis, including the mindfulness of the severity of climate change, lack of food, or the high costs of the actual systems.

Mexico relinquished its sovereignty over food policy and has shown limited capability or willingness to deal with the challenges of the food crisis in recent years. It will be necessary to adopt a more inclusive agricultural policy; the present context of uncertainty in the international grain market and negative climate effects underline the vulnerability of the food supply after these two decades of the neoliberal corn regime.

Another strategy for the Mexican State should be to rethink the businesses throughout the supply chain, which need different patterns of land use to supply consumers in local markets.

## Figures and Tables

**Table 1:** Routes and transportation means of the corn from the U.S. to Mexico.

	Nuevo Laredo	Matamoros	Veracruz
Iowa	Train: 1686 km	Train: 1800 km	Train: 220 km Water: 2300 km
Ilinois	Train: 1637 km	Train: 1700 km	Train: 405 km Water: 2300 km
Indiana	Train: 1833 km	Train: 1885 km	Train: 677 km Water: 2300 km
Total	1718 km + 650 km to the middle of the country	1795 km + 650 km to the middle of the country	434 km, train + 650 km to the middle of the country, Water: 2300 km

**Table 2:** Considered data for the impact evaluation.

Considered Data Corn for this Evaluation, in Tons			
Mexico production	1984–1993	1994–2003	2004–2013
Hybrid corn	53,157,410	76,645,180	104,478,337
Non-hybrid corn	82,738,188	109,477,191	112,175,769
Organic corn	22,521,335	29,799,691	30,534,244
Total national production	135,895,598	186,122,370	216,654,105
Imports from the U.S.	1984–1993	1994–2003	2004–2013
Hybrid corn	21,373,961	47,196,287	77,226,920
Transportation	1984–1993	1994–2003	2004–2013
Nuevo Laredo, train	15,602,991	34,453,289	56,375,651
Matamoros, train	1,496,177	3,303,740	5,405,884
Veracruz, train	4,274,792	9,439,257	15,445,383

**Table 3:** Impact results derived from total quantities of Mexican and imported corn for the last three decades of study derived from total values.

Damage Category	Unit	Corn Mexico 1984–1993	Corn Mexico 1994–2003	Corn Mexico 2004–2013	Corn U.S. 1984–2013	Corn U.S. 1994–2003	Corn U.S. 2004–2013
Human Health	DALY	178,431	244,175	28,3426	29,815	65,837	107,729
Ecosystems	species.year	3085.64	4206.83	4821.37	426.53	941.87	1541.18
Resources	$	2,723,802,639	3,739,806,116	4,389,726,449	494,797,850	1,092,615,886	1,787,828,511
Total	GPt	8.60	11.78	13.70	1.44	3.19	5.22
Human Health	GPt	3.92	5.36	6.23	0.65	1.44	2.36
Ecosystems	GPt	1.34	1.83	2.10	0.18	0.41	0.67
Resources	GPt	3.33	4.57	5.37	0.60	1.33	2.18

**Table 4:** Results by functional unit.

Damage Category	Unit	Corn Mex. 1984–1993	Corn Mex. 1994–2003	Corn Mex. 2004–2013	Corn U.S. 1984–2013
Human health	DALYs	1313.00	1311.90	1308.19	1394.99
Ecosystems	species.year	22.70	22.60	22.25	19.95
Resources	$	20,043,435	20,093,305	20,261,460	23,150,604
Total	MPt	63.30	63.29	63.26	67.71
Human health	MPt	28.87	28.84	28.76	30.67
Ecosystems	MPt	9.89	9.85	9.70	8.70
Resources	MPt	24.54	24.60	24.80	28.33

## Appendix A

**Table A1:** Produced Corn Grain by Federal Entity in Mexico 1984–2013.

National Volume of Produced Corn Grain by Federal Entity													
(tons)													
Country of Origin	1984	1985	1986	1987	1988	1989	1990	1991	1992	1993	1994	1995	1996
Total	12,788,809	14,103,454	11,909,708	11,606,945	10,592,291	10,952,847	14,635,439	14,251,500	16,929,342	18,125,263	18,235,826	18,352,856	18,025,952
Aguascalientes	51,336	45,083	46,410	55,838	47,108	41,799	74,222	47,420	73,188	65,997	74,037	85,562	77,249
Baja California	6185	22,072	20,248	12,192	11,219	7670	3273	2012	25,912	61,878	23,661	6324	7176
Baja California Sur	3147	9193	3906	4600	6109	8963	26,643	77,843	87,715	89,601	97,492	40,484	85,065
Campeche	72,969	48,015	32,565	78,484	12,839	55,907	92,766	55,565	111,122	82,268	115,314	54,889	133,041
Coahuila de Zaragoza	39,219	33,836	34,418	42,798	45,822	30,552	46,408	62,955	130,403	104,002	96,172	44,855	31,851
Colima	100,825	67,094	50,721	47,346	69,524	66,717	75,270	65,372	58,119	76,546	90,568	90,654	94,318
Chiapas	1,195,663	1,460,524	1,387,228	1,119,747	1,067,807	1,125,677	1,075,348	983,415	1,607,369	1,594,100	1,096,254	1,696,001	1,543,675
Chihuahua	259,440	357,368	320,194	399,118	250,425	235,504	435,729	739,955	948,238	880,082	487,031	303,627	412,303
Distrito Federal	32,429	27,155	34,728	28,774	25,152	28,642	21,786	22,168	16,565	16,070	16,216	12,826	12,758
Durango	152,793	267,759	221,839	204,004	168,950	109,676	234,458	239,127	248,521	289,215	325,088	291,280	288,146
Guanajuato	507,962	505,636	519,230	434,198	410,681	408,406	666,431	532,760	784,174	1,255,706	1,020,245	824,005	757,368
Guerrero	718,093	814,860	537,017	778,936	863,892	972,546	828,356	786,516	983,801	886,836	765,736	1,112,254	1,072,124
Hidalgo	306,789	394,979	307,638	328,773	336,414	358,045	439,723	383,867	485,430	362,081	453,166	406,140	427,970
Jalisco	2,031,745	2,040,200	1,857,714	1,768,973	1,812,271	1,534,645	2,226,388	2,310,590	2,421,193	2,379,659	2,125,336	2,231,290	2,328,157
México	2,163,636	2,310,927	2,033,605	1,886,116	617,405	1,179,515	2,397,144	1,755,997	1,901,215	1,233,450	1,561,746	2,146,471	2,250,753
Michoacán de Ocampo	724,483	875,444	857,165	840,501	842,049	644,091	904,757	979,195	920,566	1,060,769	1,042,268	1,293,058	1,130,533
Morelos	97,354	62,514	52,479	43,873	65,681	87,317	95,854	67,511	102,929	94,753	97,599	115,943	100,732
Nayarit	148,649	150,707	140,110	99,731	142,313	141,410	144,399	177,992	170,805	181,366	317,063	225,790	224,996
Nuevo León	61,087	50,671	44,618	68,537	50,947	38,246	61,180	91,140	92,629	99,691	159,112	54,759	43,347
Oaxaca	450,587	487,810	285,125	350,990	509,867	542,039	452,964	422,014	512,818	547,654	623,953	720,714	683,624
Puebla	967,772	1,016,617	487,122	563,426	570,023	897,753	1,077,138	1,020,398	1,164,429	1,018,884	881,146	1,063,857	1,182,504
Querétaro	108,051	136,846	89,024	76,410	44,790	94,352	107,156	60,640	136,505	111,856	168,409	186,173	169,207
Quintana Roo	22,209	8433	20,977	30,843	6045	21,669	34,370	16,227	33,546	16,848	6616	10,410	37,778
San Luis Potosí	134,760	163,358	112,205	163,037	203,573	146,417	197,093	210,361	174,692	135,392	193,209	160,989	169,285
Sinaloa	137,995	222,854	139,692	149,821	140,383	237,518	317,517	821,000	960,109	2,449,096	2,762,275	2,027,474	1,696,177
Sonora	90,198	189,506	287,617	111,219	192,990	37,355	119,401	393,714	291,271	456,659	542,981	457,480	836,442
Tabasco	75,890	93,495	85,822	102,224	75,719	82,661	92,162	74,294	67,025	71,205	125,365	99,995	140,937
Tamaulipas	735,306	755,793	740,549	441,571	893,661	543,603	658,631	443,304	747,037	1,108,758	1,355,550	818,609	230,338
Tlaxcala	307,823	337,624	187,905	301,219	119,982	275,538	305,474	262,051	379,671	253,806	310,065	297,076	328,046
Veracruz de Ignacio de la Llave	611,173	757,809	572,921	569,513	700,055	719,787	846,122	797,570	895,397	779,912	929,953	1,104,281	1,182,712
Yucatán	116,069	93,528	104,124	120,941	8924	87,651	118,860	131,844	153,048	116,297	94,582	73,136	45,049
Zacatecas	357,172	295,744	294,792	383,192	279,671	191,176	458,416	216,683	243,900	244,826	277,618	296,450	302,291
**National Volume of Produced Corn Grain by Federal Entity**													
(tons)													
Country of origin	1997	1998	1999	2000	2001	2002	2003	2004	2005	2006	2007	2008	2009
Total	17,656,258	18,454,710	17,706,376	17,556,905	20,134,312	19,297,755	20,701,420	21,685,833	19,338,713	21,893,209	23,512,752	24,410,279	20,142,816
Aguascalientes	63,082	91,494	37,657	31,692	42,942	58,240	58,684	49,059	41,520	51,318	47,305	83,804	45,404
Baja California	6704	8464	6848	9917	5235	6069	4209	25	540	86	0	0	0
Baja California Sur	88,657	54,036	56,751	37,279	32,489	29,398	30,202	38,301	28,122	26,238	27,332	28,051	20,716
Campeche	189,481	223,210	238,618	251,763	199,672	31,383	189,815	272,186	361,606	314,082	164,040	225,492	278,698
Coahuila de Zaragoza	49,231	41,265	18,376	28,226	23,247	21,619	32,108	50,849	18,412	21,910	23,953	23,305	16,507
Colima	70,194	57,860	54,696	37,680	42,148	37,416	29,723	35,963	37,521	31,193	43,403	39,633	28,733
Chiapas	1,319,230	1,755,859	2,135,550	1,887,370	1,754,130	1,858,328	2,002,592	1,353,159	1,402,833	1,592,174	1,525,578	1,625,350	1,218,456
Chihuahua	768,249	572,755	498,255	453,483	657,452	557,963	531,684	745,696	671,479	678,609	848,566	829,905	974,936
Distrito Federal	15,211	9547	12,998	12,071	12,654	10,566	9492	9411	5937	8096	9467	8378	7964
Durango	238,427	213,029	165,860	173,139	192,313	256,838	402,644	374,632	254,961	342,149	290,317	310,877	334,089
Guanajuato	558,237	993,742	582,434	652,661	1,242,638	1,189,770	1,261,338	1,638,580	1,037,035	1,068,067	1,374,287	1,499,194	844,470
Guerrero	812,128	1,132,220	1,269,519	1,181,463	1,038,965	919,054	1,209,164	1,146,194	1,195,169	1,215,411	1,304,263	1,403,046	1,135,837
Hidalgo	465,226	502,203	526,650	595,979	607,912	578,168	604,208	618,153	561,490	649,211	590,510	627,557	513,060
Jalisco	2,074,466	2,782,997	2,482,087	2,158,926	2,888,963	3,061,055	3,122,596	3,351,592	2,620,010	3,030,254	3,251,675	3,205,017	2,543,056
México	2,309,408	1,591,534	2,193,507	1,757,710	2,284,682	1,976,788	1,923,410	1,680,872	1,211,436	1,801,331	2,002,701	1,902,019	1,316,202
Michoacán de Ocampo	985,172	1,151,332	1,383,741	1,103,374	1,333,354	1,304,269	1,442,715	1,267,501	1,309,695	1,405,551	1,566,712	1,608,916	1,182,458
Morelos	98,534	99,590	90,723	83,719	122,714	55,805	84,902	83,965	84,419	91,499	102,470	94,604	85,315
Nayarit	242,120	234,902	212,157	226,525	200,519	198,328	184,961	204,071	124,680	176,858	227,780	186,568	214,440
Nuevo León	64,558	25,294	33,174	31,083	32,861	54,789	52,898	70,312	71,147	35,192	59,419	30,373	35,932
Oaxaca	625,270	735,693	741,920	817,497	804,897	601,083	713,743	694,116	601,228	627,866	766,994	785,594	594,932
Puebla	797,162	790,027	861,374	925,136	1,121,841	724,907	863,243	855,354	777,757	1,016,585	942,316	1,020,642	658,118
Querétaro	156,342	233,036	143,491	176,975	274,922	308,707	285,928	307,361	202,051	189,430	376,460	311,989	214,761
Quintana Roo	49,731	44,869	53,324	34,318	38,593	17,082	58,127	16,782	36,381	48,504	15,692	4160	33,770
San Luis Potosí	177,986	192,227	124,474	128,780	140,819	151,451	188,859	185,658	169,720	162,991	174,875	218,560	114,075
Sinaloa	2,700,843	2,618,852	1,476,451	2,319,475	2,650,714	3,149,995	2,741,316	4,004,140	4,192,846	4,398,420	5,132,809	5,368,862	5,236,720
Sonora	641,000	330,914	307,366	69,763	77,510	149,032	229,058	75,989	119,533	186,656	143,891	176,888	103,488
Tabasco	154,920	107,359	140,280	159,851	179,105	160,023	145,921	150,828	102,161	126,382	91,937	124,105	117,534
Tamaulipas	262,694	344,123	303,683	281,042	153,361	194,527	290,145	518,876	711,304	682,923	632,825	555,825	428,198
Tlaxcala	178,806	176,119	150,426	279,614	312,696	171,276	265,991	292,186	189,863	267,134	287,555	311,568	274,416
Veracruz de Ignacio de la Llave	1,121,082	947,968	1,040,815	1,242,284	1,216,357	1,080,540	1,095,484	1,052,571	888,843	1,097,405	966,463	1,330,345	1,138,875
Yucatán	142,088	117,848	159,698	160,737	129,598	12,664	123,481	128,483	108,612	146,319	139,258	27,790	44,221
Zacatecas	230,019	274,344	203,475	247,373	319,008	370,623	522,779	412,969	200,401	403,365	381,899	441,862	387,437
**National Volume of Produced Corn Grain by Federal Entity**				
(tons)				
Country of origin	2010	2011	2012	2013
Total	23,301,878	17,635,417	22,069,254	22,663,953
Aguascalientes	51,630	51,247	56,307	79,956
Baja California	0	0	6	1472
Baja California Sur	17,095	19,546	32,056	21,288
Campeche	384,582	457,009	343,904	440,546
Coahuila de Zaragoza	39,278	9750	13,153	40,122
Colima	38,141	39,912	33,705	38,156
Chiapas	1,394,496	1,554,368	1,404,680	1,529,385
Chihuahua	1,068,689	851,208	1,113,012	1,309,634
Distrito Federal	8829	4881	5521	5251
Durango	249,437	177,148	211,489	297,383
Guanajuato	1,185,172	1,015,660	1,217,706	1,526,682
Guerrero	1,413,973	1,309,068	1,304,133	989,673
Hidalgo	613,320	454,945	704,422	644,628
Jalisco	3,395,072	2,519,276	3,235,189	3,303,498
México	1,549,545	649,179	1,575,300	2,012,774
Michoacán de Ocampo	1,526,484	1,386,363	1,801,965	1,746,768
Morelos	94,008	89,885	86,479	80,499
Nayarit	176,224	188,046	201,138	193,606
Nuevo León	60,735	20,148	61,415	99,733
Oaxaca	645,531	694,554	729,351	628,530
Puebla	1,080,462	611,805	1,002,278	942,171
Querétaro	282,427	158,895	272,414	301,607
Quintana Roo	55,779	67,470	54,363	70,491
San Luis Potosí	165,768	112,908	105,381	154,150
Sinaloa	5,227,872	2,929,180	3,646,875	3,627,778
Sonora	240,954	83,221	167,125	113,534
Tabasco	104,467	132,708	181,557	155,183
Tamaulipas	540,170	489,380	517,670	400,057
Tlaxcala	305,544	158,685	313,879	284,118
Veracruz de Ignacio de la Llave	973,458	1,039,846	1,275,318	1,192,169
Yucatán	120,542	149,060	113,380	103,914
Zacatecas	292,195	210,067	288,081	329,200

Source: SIAP with delegation information from SAGARPA.

## Appendix B

**Table B1:** Analysis by Functional Unit.

Functional Unit: 1 Million Tons of Corn		
Mexican production during the period 1984–1993		
53.15 million ton	39.11%	Hybrid corn
60.21 million ton	44.33%	Non-hybrid corn with chemicals
22.52 million ton	16.59%	Organic corn
135.83 million ton	100%	Total
Transportation and production results within of Mexico by functional unit		
1.00 E8 ton-km	Bus average fuel mix	Non-hybrid corn with chemicals
443.11E6 ton-km	Train fuel mix 80%	Hybrid corn
Production		
391,100 ton	Corn grain US	Hybrid corn
443,000 ton	Corn grain ROW	Non-hybrid corn with chemicals
165,000 ton	Corn grain, organic	Organic corn
Mexican Production during the period of 1994–2003		
76.645 million ton	41.18%	Hybrid corn
79.667 million ton	42.80%	Non-hybrid corn with chemicals
29.799 million ton	16.02%	Organic corn
186.122 million ton	100%	Total
Transportation and production results inside of Mexico by functional unit		
1.00 E8 ton-km	Bus average fuel mix	Non-hybrid corn with chemicals
466.57E6 ton-km	Train fuel mix 80%	Hybrid corn
Production		
411,800 ton	Corn grain U.S.	Hybrid corn
428,000 ton	Corn grain ROW	Non-hybrid corn with chemicals
160,200 ton	Corn grain, organic	Organic corn
Mexican Production during the period of 2004–2013		
104.478 million ton	48.22%	Hybrid corn
81.641 million ton	37.68%	Non-hybrid corn with chemicals
30.534 million ton	14.1%	Organic corn
216.654 million ton	100%	Total
Transportation and production results within of Mexico by functional unit		
1.00 E8 ton-km	Bus average fuel mix	Non-hybrid corn with chemicals
546.332E6 ton-km	Train fuel mix 80%	Hybrid corn
Production		
482,200 ton	Corn grain U.S.	Hybrid corn
376,800 ton	Corn grain ROW	Non-hybrid corn with chemicals
141,000 ton	Corn grain, organic	Organic corn
Functional Unit: 1 Million of tons of imported corn from EEUU		
Derived Mexican production from the period of 1984–2013 of hybrid corn		
Nuevo Laredo	73%	Hybrid corn
Matamoros	7%	Hybrid corn
Veracruz	20%	Hybrid corn
Total	100%	
US Transportation and production results by functional unit		
1.986E9 ton-km	Diesel train powered/U.S.	Hybrid corn
460E6 ton-km	Barge average fuel/U.S.	Hybrid corn
Production		
1E6 ton	Maice grain U.S.	Hybrid corn
